# Multiple-site decontamination to prevent acquired infection in patients with veno-venous ECMO support

**DOI:** 10.1186/s13613-023-01120-1

**Published:** 2023-04-07

**Authors:** Nicolas Massart, Christophe Camus, Nicolas Nesseler, Pierre Fillâtre, Erwan Flecher, Alexandre Mansour, Jean-Philippe Verhoye, Lucie Le Fevre, Charles-Edouard Luyt

**Affiliations:** 1 Service de Réanimation, CH de St BRIEUC, 10, rue Marcel Proust, 22000 Saint-Brieuc, France; 2 Service de réanimation médicale, CHU de Rennes, 2, rue Henri le Guilloux, 35000 Rennes, France; 3 Department of Anesthesia and Critical Care, Rennes University Hospital, Rennes, France; 4 Univ Rennes, CHU de Rennes, Inra, Inserm, Institut NUMECAN – UMR_A 1341, UMR_S 1241, CIC 1414 (Centre d’Investigation Clinique de Rennes), 35000 Rennes, France; 5 Department of Thoracic and Cardiovascular Surgery, Rennes University Hospital, University of Rennes 1, Signal and Image Treatment Laboratory (LTSI), Inserm U1099, Rennes, France; 6 Service de Médecine Intensive Réanimation, Institut de Cardiologie, Assistance Publique–Hôpitaux de Paris (APHP), Paris, France; 7 Sorbonne-Université, Hôpital Pitié–Salpêtrière, and Sorbonne Université, INSERM, UMRS_1166-ICAN Institute of Cardiometabolism and Nutrition, Paris, France

**Keywords:** Critical care, Pneumonia, Bacteremia, Hospital acquired infection, Extra corporal membrane oxygenation

## Abstract

**Background:**

Acute distress respiratory syndrome (ARDS) patients with veno-venous extra corporeal membrane oxygenation (ECMO) support are particularly exposed to ECMO-associated infection (ECMO-AI). Unfortunately, data regarding AI prophylaxis in this setting are lacking. Selective decontamination regimens decrease AI incidence, including ventilator-associated pneumonia (VAP) and bloodstream infection (BSI) in critically ill patients. We hypothesized that a multiple-site decontamination (MSD) regimen is associated with a reduction in the incidence of AI among VV-ECMO patients.

**Methods:**

We conducted a retrospective observational study in three French ECMO referral centers from January 2010 to December 2021. All adult patients (> 18 years old) who received VV-ECMO support for ARDS were eligible. In addition to standard care (SC), 2 ICUs used MSD, which consists of the administration of topical antibiotics four times daily in the oropharynx and the gastric tube, once daily chlorhexidine body-wash and a 5-day nasal mupirocin course. AIs were compared between the 2 ICUs using MSD (MSD group) and the last ICU using SC.

**Results:**

They were 241 patients available for the study. Sixty-nine were admitted in an ICU that applied MSD while the 172 others received standard care and constituted the SC group.

There were 19 ECMO-AIs (12 VAP, 7 BSI) in the MSD group (1162 ECMO-days) compared to 143 AIs (104 VAP, 39 BSI) in the SC group (2376 ECMO-days), (*p* < 0.05 for all infection site). In a Poisson regression model, MSD was independently associated with a lower incidence of ECMO-AI (IRR = 0.42, 95% CI [0.23–0.60] *p* < 0.001). There were 30 multidrug resistant microorganisms (MDRO) acquisition in the SC group as compared with two in the MSD group (IRR = 0.13, 95% CI [0.03–0.56] *p* = 0.001). Mortality in ICU was similar in both groups (43% in the SC group vs 45% in the MSD group *p* = 0.90). Results were similar after propensity-score matching.

**Conclusion:**

In this cohort of patients from different hospitals, MSD appeared to be safe in ECMO patients and may be associated with improved outcomes including lower ECMO-AI and MDRO acquisition incidences. Since residual confounders may persist, these promising results deserve confirmation by randomized controlled trials.

**Supplementary Information:**

The online version contains supplementary material available at 10.1186/s13613-023-01120-1.

## Background

For the more severe acute distress respiratory syndrome (ARDS) patients, veno-venous extra corporeal membrane oxygenation (VV-ECMO) support is a life saving therapy [1]. Unfortunately, because of associated conditions and ECMO-induced immune dysfunction, up to 55% of patients will subsequently develop an acquired infection during ECMO support (ECMO-AI) [2–6] with implication for mortality [2, 7].

Selective decontamination regimens are infection prevention measures that decreases AI, including ventilator-associated pneumonia (VAP) and bloodstream infection (BSI) and may decrease mortality in specific population [8–11]. Recently, multiple-site decontamination (MSD) which consists of a combination of topical antibiotic in the oropharyngeal and digestive tract with chlorhexidine body-wash and intra-nasal mupirocin have shown favorable results on VAP, BSI and mortality in ARDS patients [10–12]. However, data regarding its effect on the more severe cases with VV-ECMO support are lacking. We hypothesized that MSD was associated with a reduction in the incidence of ECMO-AI among VV-ECMO patients.

## Methods

### Setting and patients

We conducted a retrospective observational study in three French ECMO referral centers in the medical Intensive Care unit (ICU) of Rennes University Hospital, the medical ICU of La Pitié Salpêtrière in Paris and the polyvalent ICU of Saint-Brieuc. Patients were screened from each hospital from respective ECMO cohorts and all adult patients (> 18 year old) who received VV-ECMO support for ARDS were eligible. In La Pitié Salpétrière, the cohort was constituted from 1st January 2017 until 31st December 2019, whereas in the other ICUs, patients were included from 1st January 2010 to 31st December 2021. Those who received VA-ECMO only and those who were already included in the database during a previous VV-ECMO run were excluded. Patients with liberty deprivation, and patients younger than 18 years were excluded from the study.

### Intervention

In addition to standard care (SC), MSD was applied in patients admitted in Rennes and Saint-Brieuc ICU. This strategy is part of the local protocol for the prevention of acquired infections and is systematically applied in intubated patients [12]. MSD is a variant of selective digestive decontamination, and consists of the administration of (i) topical antibiotics including an aminoglycoside (tobramycin, 300 mg per day, in Rennes or gentamicin, 543 mg per day, in Saint-Brieuc), colistin sulfate (400 mg per day) and amphotericin B (2 g per day), four times daily in the oropharynx and the gastric tube, (ii) 4% chlorhexidine body washing daily and (iii) 5-day nasal mupirocin course. MSD do not include a systematic intravenous antimicrobial agent as part of decontamination regimen. In Rennes and Saint-Brieuc ICUs, MSD was systematically applied in all patients who had an expected intubation duration of 24 h or more throughout the duration of intubation. Full details about the MSD regimen have been reported elsewhere [10–12]. Patients in La Pitié Salpêtrière medical ICU received standard care alone.

### AI prevention measures

In all ICUs, VAP prevention measure consisted of a bundle of care that included semi-recumbent positioning (at a maximum of 30°, and depending on its feasibility and tolerance in patients having femoral cannula), specific oral care with tooth brushing and mouth washing every 6 h and subglottic secretion drainage every 4 h. Topical antibiotic, chlorhexidine body washing and mupirocin were not used in La Pitié-Salpêtrière patients. Mouth washing was performed with 0.2% chlorhexidine in La Pitié-Salpêtrière and Saint-Brieuc ICUs but not in Rennes ICU. Subglottic secretion drainage was performed in La Pitié-Salpêtrière patients every 4 h but not in Rennes and St Brieuc.

In La Pité-Salpêtrière hospital, catheter dressing (central venous and arterial lines) were performed using chlorhexidine-impregnated dressing, except for the first 24–48 h, and changed every 7 days or sooner in case of bleeding. Dressings were performed with dry sterile compresses in Rennes and St Brieuc ICUs and were changed weekly or sooner in case of bleeding. Adherence to these measures was not retrieved in the present report. There were no systematic antibioprophylaxis during cannulation in any centers.

### VV-ECMO management

ECMO management in the three ICUs is reported elsewhere [13, 14]. Briefly, VV-ECMO was inserted percutaneously through right femoral (drainage) and right jugular (reinjection) veins. Ultra-sonography guided Seldinger technique was applied for vascular access while chest ultra-sonography and X-ray confirmed intra-thoracic placement. Intravenous unfractioned heparin bolus was administrated unless contra-indications and was followed with continuous administration unless bleeding or treatment-related complications. A nurse-directed sedation and analgesia protocol was routinely used in the three ICUs. Using Richmond Agitation–Sedation Scale (RASS) and Behavioral Pain Scale (BPS), nurses recorded agitation and pain levels hourly and titrated infusion to obtain prescribed targets (RASS between [–1, 0] and BPS < 4).The sedative and analgesic drug choice was at the clinician’s discretion. The weaning procedure was also similar in the three ICUs. Nurse-to-patient ratio was different between the 3 ICUs: 1:2.5 in Rennes and St Brieuc and 1:2 in La Pitié-Salpêtrière.

### Definition

ECMO-AI was considered when the infection developed during VV-ECMO run and was diagnosed 48 h or more after admission and was not incubating on admission. Diagnosis was made by treating physician. BSI was defined as a positive blood culture unless for common skin contaminants requiring 2 positives blood cultures drawn on separate occasions [15]. VAP diagnosis relied on clinical signs (fever, purulent sputum, hypoxia), radiological findings (new infiltrate on chest X-ray or CT scan), and leukocytosis in a patients intubated for more than 48 h [16]. All VAP were bacteriologically confirmed. Respiratory samples for VAP diagnosis were performed either using fiberoptic broncho-alveolar lavage or endotracheal aspiration, according to local practices. Threshold for lung samples positivity were 10^4^ cfu/mL for BAL and 10^5^ cfu/mL for tracheal aspirate. Each center had a nosocomial infection committee for the prevention and prospective census of acquired infections and applied the recommendations of the French Society for Hospital Hygiene for the prevention and treatment of infection (available at https://sf2h.net/publications/actualisation-precautions-standard-2017). Microorganisms responsible for infection were considered as multidrug resistant according to the European Society of Clinical Microbiology and Infectious Disease definition [17]. In all participating ICUs, patients were screened for multidrug-resistant organisms (MDRO) rectal carriage at admission, weekly afterwards and at discharge on rectal swabs. As described elsewhere, patients with no prior colonization (no colonization at admission) who tested positive for MDRO on either rectal screening or on a blood or respiratory sample were considered as having MDRO acquisition [18].

### Primary and secondary endpoints

The primary endpoint was the incidence of ECMO-AI, and secondary endpoints were antimicrobial agent consumption for treatment of AI, specific ECMO-associated VAP and BSI incidences, MDRO acquisition, as well as survival during ECMO support.

Antimicrobial agent consumption was calculated for each patient and corresponds to the number of day with antimicrobial treatment. Then, the number of days alive and without antimicrobial treatment during the 60 days following cannulation was also reported.

### Ethical statement

Patients or closest relative were informed of the anonymous prospective collection of their data for constitution of a cohort and had the possibility not to participate in the study. In case of refusal, the data were not collected accordingly. This manuscript follows the STROBE statement for reporting cohort studies. The study protocol received approval from the ethical committee of the French Intensive Care Society (CE 21-14).

### Statistical analysis

Statistical analysis was performed with the statistical software R 4.1.1. Categorical variables were expressed as percentages and continuous variables as median and interquartile range (IQR). The Chi-square test and Fisher exact test were used to compare categorical variables and the Man–Whitney U test or the Wilcoxon for continuous variables.

Risk factors for ECMO-AI and death were estimated using bivariate and multivariable logistic regression and Kaplan–Meier survival curves with log rank test were used for survival analysis. Multivariable analyses were performed with inclusion of non-redundant variables associated with event (ECMO AI or death) with a *p*-value < 0.2 in the bivariate analysis. Overall, 11.8% of the data were missing (172 patients had at least one missing data). For the purpose of the multivariable analysis, missing data were considered as missing at random and were handled using chained equation, using “MICE” R package to create an imputed dataset. Incidence rate were compared using a Poisson regression model. Finally, to draw unbiased marginal estimates of exposure effect, a propensity-score matched analysis was performed. Propensity score was calculated using a non-parsimonious logistic regression model including all baseline variables listed in Table 1 (i.e., age, sex, comorbidities, period of admission, ARDS etiology, MDRO colonization at admission, non-respiratory SOFA score at cannulation, PaO2/FiO2 ratio at cannulation, PaCO2 at cannulation and lactates at cannulation). Then, using the “MatchIt” package, a *k*-nearest neighbor algorithm was used for propensity-score matching with a 1:1 ratio. The balance between matched groups was evaluated by the analysis of the standardized mean differences after matching. A post-matching difference < 0.2 was considered as an acceptable bias reduction. All tests were two-sided, and *p* < 0.05 was considered statistically significant.

**Table 1:** Baseline characteristics and outcomes of study patients

Variables	Missing data	SC	MSD	*p*-value
	SC/MSD	*n* = 172	*n* = 69	
Age, year	0/0	51 [39–61]	46 [34–64]	0.722
Male—no. (%)	0/1	108 (62.8)	47 (68.1)	0.528
Immunocompromised—no. (%)	0/0	47 (27.3)	14 (20.3)	0.331
Onco-hematological malignancies—no. (%)	0/0	23 (13.4)	9 (13.0)	1.000
Immunomodulatory treatment—no. (%)	0/0	19 (11.0)	3 (4.3)	0.166
Other immunodepression—no. (%)	0/0	7 (4.1)	1 (1.4)	0.529
Diabetes—no. (%)	0/0	34 (19.8)	7 (10.1)	0.108
Artery disease—no. (%)	0/0	3 (1.7)	1 (1.4)	1.000
Hypertension—no. (%)	0/0	54 (31.4)	11 (15.9)	0.022
Period of admission	0/0			0.610
< 2018—no. (%)		112 (65.1)	48 (69.5)	
2018–2021—no. (%)		60 (34.9)	21 (30.4)	
ARDS etiology	0/0			0.032
Others—no. (%)		82 (47.7)	29 (42.0)	
Bacterial—no. (%)		63 (36.6)	19 (27.5)	
Viral—no. (%)		27 (15.7)	21 (30.4)	
MDRO colonization at admission—no. (%)	6/0	22 (13.5)	6 (8.7)	0.420
ESBL-PE– no. (%)		20 (90.9)	4 (66.7)	
Carabapenem-resistant *enterobacteriaceae*—no. (%)		1 (4.5)	0 (0.0)	
Methicillin-resistant *Staphylococcus aureus*—no. (%)		0 (0.0)	1 (16.7)	
Imipenem-resistant *Acinetobacter baumannii*—no. (%)		1 (4.5)	0 (0.0)	
Multidrug-resistant *Pseudomonas aeruginosa*—no. (%)		0 (0.0)	1 (16.7)	
SOFA score at cannulation,	2/0	14 [10–17]	9 [7–11]	< 0.001
PaO2/FiO2 at cannulation, mmHg	57/4	60 [54–78]	72.40 [55.50–99.50]	0.015
PaCO2 at cannulation, mmHg	118/6	61 [48.25–77.50]	51 [41–64.25]	0.017
Lactate at cannulation, mmol/L	143/14	5.30 [2.30–8.00]	2.40 [1.50–3.25]	0.005
Outcomes				
ECMO-associated infection—no. (%)	0/0	67 (39.0)	13 (18.8)	0.004
Ventilator-associated pneumonia—no. (%)	0/0	58 (33.7)	12 (17.4)	0.018
Bloodstream infection—no. (%)	0/0	31 (18.0)	7 (10.1)	0.186
Number of days with antimicrobial treatment, days	0/10	13 [5–30]	7 [2–10]	< 0.001
Number of days alive without antimicrobial treatment at day 60	0/10	27 [0–53]	42 [8–53]	0.009
Length of ECMO support, days	0/3	7 [4–18]	11 [5–24]	0.234
Length of ICU stay, days	60/1	22 [10–40]	24 [15–42]	0.427
MDRO colonization acquisition—no. (%)	8/0	30 (18.3)	2 (2.9)	0.004
Death during ECMO support—no. (%)	0/0	64 (37.2)	26 (37.7)	1.000
Death in ICU—no. (%)	0/0	74 (43.0)	31 (44.9)	0.90

SC: standard care; MSD: multiple-site decontamination; ARDS: acute respiratory distress syndrome; SOFA: Sequential Organ Failure Assessment; MDRO: multidrug-resistant microorganisms; ESBL-PE: extended spectrum beta lactamase producing *Enterobacteriaceae*; ICU: intensive care unit; ECMO: extra corporeal membrane oxygenation; AI: acquired infection

## Results

### Population

**Fig. 1:**
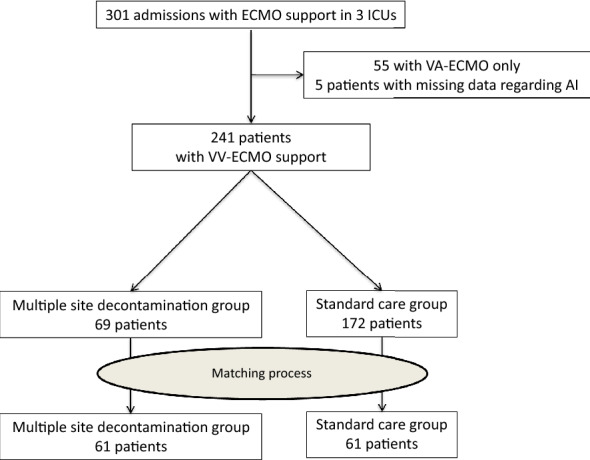
Flowchart

They were 301 patients with ECMO support included in the cohorts of the 3 participating ICUs. Among them, 55 received VA-ECMO only and 5 had missing data regarding ECMO-AI leaving 241 patients finally available for the study. Sixty-nine were admitted in an ICU that applied MSD and constituted the MSD group while the 172 others received standard care and constituted the SC group (Fig. 1). The mean annual MDRO prevalence rate in participating ICUs was 6.8% [6.3–7.2]) and tended to be lower in ICUs that applied MSD (6.3% [5.7–7.2]) as compared with the ICU that applied SC (10.2% [8.6–11.1], *p* = 0.043). The mean annual hydro-alcoholic solution consumption in participating ICUs was 106 L per 1000 patients-days [97–140], and was higher in the ICU that applied SC (180 L per 1000 patients-days [165–210]) as compared with ICUs that applied MSD (104 L per 1000 patients-days [96–108], *p* < 0.001).

At admission, age was 51 years [37–61], 25% of the patients were immunocompromised, SOFA score at cannulation was 12 [9–16], PaO2/FiO2 ratio on the day of cannulation was 64 mmHg [54–83] (Table 1). Baseline characteristics were not balanced between groups with a lower proportion of patients with hypertension, more patients cannulated for viral ARDS, a lower SOFA score, a higher PaO2/FiO2 ratio, a lower PaCO2 and a lower lactate level in the MSD group as compared with SC group.

### Acquired infections and colonization

There were 19 ECMO-AIs (12 VAP, 7 BSI) during the 1162 ECMO-days in the MSD group as compare with 143 AIs (104 VAP, 39 BSI) in 2376 ECMO-days in the SC group, (incidence rate ratio [IRR] = 0.26, 95% CI [0.16–0.42], *p* < 0.001) (Table 1). Similarly, the VAP incidence rates were 10.3 per 1000 ECMO-days and 43.7 per 1000 ECMO-days, respectively (IRR = 0.23, 95% CI 0.13–0.41, *p* < 0.001). The BSI incidence rate was also lower in the MSD group, with incidence rates of 6.0 and 16.4 per 1000 ECMO-days, respectively (IRR = 0.36, 95% CI [0.16–0.81], *p* = 0.010).

In a Poisson regression model, MSD was independently associated with a lower incidence of ECMO-AI (IRR = 0.42, 95% CI [0.23–0.60] *p* < 0.001) (Table 2), of VAP (IRR = 0.31, 95% CI [0.17–0.55] *p* < 0.001) but not of BSI (IRR = 0.58, 95% CI [0.24–1.38] *p* = 0.216). More recent year of cannulation was associated with an increased risk of AI, whereas bacterial or other etiology of ARDS was associated with a lower rate of AI as compared with viral cause of ARDS.

**Table 2:** Risk factors for ICU acquired infection (Poisson regression)

Variables	IRR	95% CI	*p*-value	IRR	95% CI	*p*-value
Multiple-site decontamination	0.37	0.24–0.57	< 0.001	0.40	0.24–0.64	< 0.001
Age, per 1-year increase	1.01	1.00–1.02	0.029	1.01	1.00–1.02	0.14
Male	1.13	0.80–1.58	0.485			
Immunocompromised	0.94	0.67–1.32	0.725			
Diabetes	1.69	1.16–2.46	0.006	1.18	0.75–1.86	0.47
Artery disease	0.67	0.17–2.71	0.578			
Hypertension	1.76	1.28–2.44	< 0.001	1.26	0.84–1.89	0.26
Year of admission, per 1-year increase	1.15	1.05–1.27	0.003	1.17	1.03–1.33	0.015
ARDS etiology						
Viral	Ref	Ref	Ref	Ref	Ref	Ref
Bacterial	0.84	0.57–1.25	0.391	0.64	0.40–0.86	0.041
Others	0.68	0.47–0.98	0.041	0.59	0.42–0.98	0.007
MDRO colonization at admission	1.50	1.02–2.20	0.039	1.31	0.87–1.96	0.20
Non respiratory SOFA score, per supplementary point	1.05	1.01–1.09	0.021	1.00	0.96–1.05	0.94
PaO2/FiO2, per 1 point increment	1.02	0.99–1.05	0.057	1.00	0.99–1.00	0.23
PCO2, per 1 point increment	1.01	0.99–1.02	0.300			
Lactate, per 1 point increment	1.11	1.00–1.23	0.046	1.03	0.98–1.07	0.21

ARDS: acute respiratory distress syndrome; SOFA: Sequential Organ Failure Assessment; MDRO: multidrug-resistant microorganisms; ICU: intensive care unit; ECMO: extra corporeal membrane oxygenation

There were 30 MDRO acquisition in the SC group as compared with 2 in the MSD group (IRR = 0.13, 95% CI [0.03–0.56] *p* = 0.001).

### Microorganisms responsible for infection and antimicrobial treatment

Overall, patients in the MSD group were treated with an antimicrobial agent during 7 days [2–10] as compared with 13 days [5–30] (*p* < 0.001) in the SC group, corresponding to a number of day alive and without antimicrobial agent at day 60 of 42 days [8–53] and 27 [0–53], respectively (*p* = 0.009) (Table 1). This higher number of day alive without antimicrobial treatment was drive by more days alive without antimicrobial treatment for AI (50 days [16–60] vs 41 days [3–60] *p* = 0.012), whereas number of days alive without antimicrobial treatment for ARDS etiology was similar in between the two groups (46 days [7–55] vs 47 days [4–54], *p* = 0.289) (Additional file 1: Table S1).

Antimicrobial agents used for treatment of ARDS etiology are reported in Additional file 1: Table S1 and those used for treatment of AI are reported in Table 3. Regarding empiric therapy of AI, patients in the SC group mostly received piperacillin/tazobactam (43.1%) or carbapenems (20.7%), whereas those in the MSD group mostly received a 3rd-generation cephalosporin (38.5%) or piperacillin/tazobactam (23.1%). Regarding definitive therapy, patients in the SC group received mostly penicillin (31.0%) (14 patients treated with piperacillin, 1 with cloxacillin and 3 with amoxicillin) or an antipseudomonal cephalosporins (29.3%) while those in the DMS group received mostly a 3rd-generation cephalosporin (38.5%) or piperacillin / tazobactam (23.1%).

**Table 3:** Antimicrobial agents administrated for first acquired infection in both groups

Variables	SC	MSD	*p*-value
Empiric antimicrobial agent	*n* = 67	*n* = 13	< 0.001
Amoxicillin and clavulanic acid	1 (1.7)	0 (0.0)	
3rd-generation cephalosporin	0 (0.0)	5 (38.5)	
Anti-Pseudomonas cephalosporin	9 (15.5)	1 (7.7)	
Piperacillin/tazobactam	25 (43.1)	3 (23.1)	
Carbapanem	12 (20.7)	1 (7.7)	
Others	11 (19.0)	3 (23.1)	
Combination therapy	24 (41.4)	2 (15.4)	0.15
Second agent			< 0.001
Aminoglycoside	22 (91.7)	0 (0.0)	
Vancomycin	1 (4.2)	2 (100.0)	
Others	1 (4.2)	0 (0.0)	
Empiric antimicrobial agent appropriate	23 (34.4)	4 (30.8)	1.00

Definite antimicrobial agent			0.005
Penicillin*	18 (31.0)	0 (0.0)	
Amoxicillin and clavulanic acid	5 (8.6)	0 (0.0)	
3rd-generation cephalosporin	5 (8.6)	5 (38.5)	
Anti-Pseudomonas cephalosporin	17 (29.3)	2 (15.4)	
Piperacillin / tazobactam	4 (6.9)	3 (23.1)	
Carbapanem	4 (6.9)	0 (0.0)	
Others	5 (8.6)	3 (23.1)	
Combination therapy	8 (13.6)	0 (0.0)	0.357
Second agent			< 0.001
Aminoglycoside	2 (25.0)	0	
Colistin	4 (50.0)	0	
Fluoroquinolone	1 (12.5)	0	
Others	1 (12.5)	0	

SC: standard care; MSD: multiple-site decontamination; * there were 14 patients treated with piperacillin, 1 with cloxacillin and 3 with amoxicillin

Details regarding type of respiratory sample performed for VAP diagnosis and microorganisms responsible for AIs and MDRO acquired colonization are reported in Table 4. There was a lower proportion of non-fermenting Gram-negative bacilli (12% vs 45%, *p* = 0.029) responsible for VAP in the MSD group as compared with the SC group, but the distribution of other microorganisms responsible for AI was otherwise similar. Noteworthy, none of the four *Enterobacteriaceae* and the two non-fermenting Gram-negative bacilli responsible for AI in the DMS group were resistant to colistin.

**Table 4:** Microorganisms responsible for infections and MDRO acquired colonization

Variables	SC	MSD	*p*-value
Ventilator-associated pneumonia	*n* = 107	*n* = 16	
*Staphylococcus aureus*—no. (%)	3 (2.8)	2 (12.5)	0.126
*Streptococcus* sp.—no. (%)	2 (1.9)	1 (6.2)	0.344
*Enterococcus* sp.—no. (%)	13 (12.1)	4 (25.0)	0.235
*Enterobacteriaceae*– no. (%)	24 (22.4)	4 (25.0)	0.758
Non-fermenting Gram-negative bacilli—no. (%)	48 (44.9)	2 (12.5)	0.015
Others—no. (%)	17 (15.9)	3 (18.8)	0.724

Respiratory sample for first VAP diagnosis	*n* = 58	*n* = 12	0.36
Broncho-alveolar lavage	50 (86)	9 (75)	
Endo-tracheal aspirate	8 (14)	3 (25)	

Bloodstream infection	*n* = 40	*n* = 8	
*Staphylococcus aureus*—no. (%)	1 (2.5)	0 (0.0)	1.000
Coagulase negative *Staphylococcus*—no. (%)	4 (10.0)	0 (0.0)	1.000
*Streptococcus* sp.—no. (%)	2 (5.0)	0 (0.0)	1.000
*Enterococcus* sp.—no. (%)	11 (27.5)	5 (62.5)	0.097
*Enterobacteriaceae*—no. (%)	8 (20.0)	1 (12.5)	1.000
Non-fermenting Gram-negative bacilli—no. (%)	5 (12.5)	0 (0.0)	0.573
*Candida* sp.—no. (%)	8 (20.0)	0 (0.0)	0.320
Others—no. (%)	1 (2.5)	2 (25.0)	0.068

MDRO acquired colonization	*n* = 30	*n* = 2	
Carbapenem resistant	5 (16.7)	0 (0.0)	1.000
*Acinetobacter baumannii*—no. (%)	3 (10.0)	0 (0.0)	1.000
*Klebsiella pneumonia*—no. (%)	1 (3.3)	0 (0.0)	1.000
ESBL-PE	25 (83.3)	2 (100.0)	1.000
*Klebsiella pneumonia*—no. (%)	7 (23.3)	0 (0.0)	1.000
*Citrobacter freundii*—no. (%)	1 (3.3)	0 (0.0)	1.000
*Escherichia coli*—no. (%)	5 (16.7)	2 (100.0)	0.042
*Enterobacter cloacae*—no. (%)	12 (40.0)	0 (0.0)	0.516
Others			
MDR-* Pseudomonas aeruginosa*—no. (%)	1 (3.3)	0 (0.0)	1.000

SC: standard care. MSD: multiple-site decontamination. VAP: ventilator-associated pneumonia. MDRO: multidrug-resistant microorganisms. ESBL-PE: extended spectrum beta lactamase producing *Enterobacteriaceae*

Antimicrobial therapy for AI differed between groups (Table 4).

### Outcomes

**Fig. 2:**
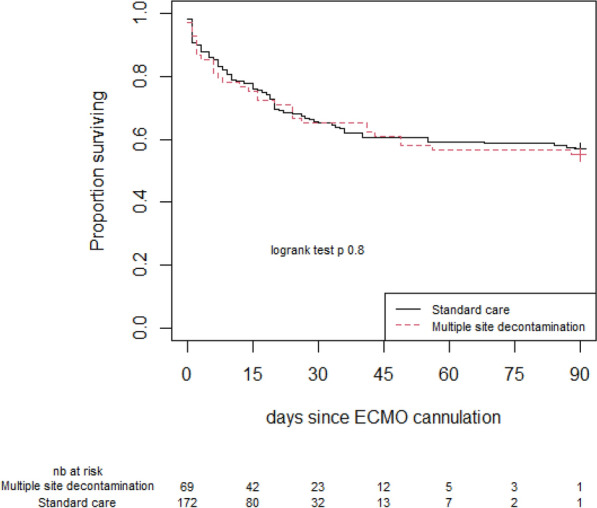
Survival curves

There were no differences in other outcomes. ECMO run lasted for 7 days [4–18] and 11 days [5–24] in the SC and MSD group, respectively (*p* = 0.234). Twenty-six patients (38%) of the MSD group died during ECMO support as compared with 64 patients (37%) in the SC group (*p* = 1.00), but finally 31 (45%) and 74 (43%) patients died in ICU, in both group, respectively (*p* = 0.90) (Table 1 and Fig. 2).

Risk factors for death in ICU are reported in Additional file 1: Table S2. MSD was not associated with death (OR = 1.08, 95% CI [0.61–1.90] *p* = 0.79), even when it was forced in the multivariable model (not shown).

### Propensity score matched analysis

**Fig. 3:**
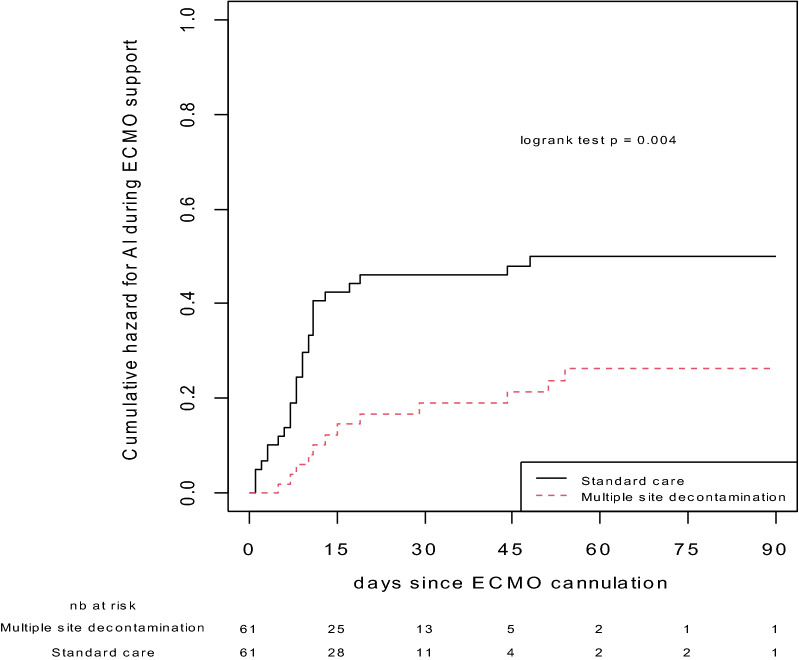
ECMO-AI cumulative incidences

As confirmatory analysis, patients were matched using a propensity-score. Matching process resulted in 61 patient’s pairs (Fig. 1 and Additional file 1: Table S3 for baseline characteristics of pairs). In this dataset, there were fewer ECMO-AI in the MSD group: 17 ECMO-AIs (11 VAP and 6 BSI) during 1062 ECMO-days versus 37 ECMO-AIs (28 VAP and 11 BSI) during 1063 ECMO-days in the SC group (IRR = 0.45 [0.25–0.80] *p* = 0.005) (*p* = 0.006 for VAP and *p* = 0.222 for BSI) (Fig. 3).

There were 2 MDRO acquisition in the MSD group as compared with 11 in the SC group (IRR = 0.18, 95% CI [0.04–0.81] *p* = 0.012).

## Discussion

In this study, we report for the first time that MSD is independently associated with a reduced rate of ECMO-AI in patients with VV-ECMO support. This favorable result was mostly driven by a reduction of VAP and was accompanied by a reduction of MDRO acquisition, but also a lower antimicrobial agent consumption.

We confirmed a high incidence rate of ECMO-AI in VV-ECMO patients as previously reported with incidence rates from 30.1 to 50.4 per 1000 ECMO-days [2, 7]. Despite ARDS patients being particularly exposed [19–21], dedicated prophylaxis in this particular setting has been poorly assessed.

For decades now, selective decontamination regimens, in addition with systematic antibiotic or not, have been associated with a reduced incidence of VAP and BSI in others critically ill populations [8–10] in ICUs with low multidrug resistance rate. Conversely, study conducted in area with higher incidence rate reported conflicting results [22]. In the present study, participating ICUs perform epidemiological watch and have well described antimicrobial resistance rate [18, 23, 24] and MDRO colonization at admission was similar in both groups. Despite high adherence to hand hygiene, the higher rate of acquired MDRO colonization in La Pitié Salpétrière led to a higher MDRO prevalence rate. VV-ECMO and ARDS patients are at high risk of MDRO acquired infection [25] with up to 56% of AIs involving MDRO [2]. A favorable result with a decrease in the incidence of MDROs, together with a decline in the consumption of broad-spectrum antimicrobials, has been reported among patients receiving selective decontamination regimens [12, 26, 27]. In the present study either, MSD was associated with a decrease of antimicrobial agent consumption, drive by a lower consumption of antimicrobial agent for treatment of AI. However, these regimens require careful evaluation in areas with intermediate-to-high MDRO rates before definite recommendations.

As a component of MSD, the role of chlorhexidine bathing is uncertain. In areas with moderate-to-high MDRO prevalence rate, cutaneous chlorhexidine washing significantly reduces the risks of acquisition of MDROs and development of hospital-acquired bloodstream infections [28]. Conversely, in a randomized controlled trial conducted in France, chlorhexidine body-wash (combined with intra-nasal mupirocin) did not significantly reduce AI compared to placebo [10]. Interestingly, mupirocin/chlorhexidine in combination with oropharyngeal and digestive decontamination (MSD) produced a synergic preventive effect, with favorable results reported on either overall AIs, VAP, BSI, MDRO acquisition [10, 11, 26, 29]. ICU mortality was also lower with MSD than in the control group in several studies [10, 11, 29].

Attributable mortality of VAP is a matter of debate. Melsen et al. observed that the patients with moderate range severity are those with the higher VAP attributable mortality. In contrast, the outcome of those with low and high range severity, such as patients with VV-ECMO support, is minimally affected by VAP onset [30]. Conversely, an increased mortality has been previously described in patients with ECMO-AI, especially in those with VAP, but remaining confounders should be acknowledged [2, 31]. Indeed, VAP occurs in the more severe patients and especially those with persistent organ failure despite support. Finally, death may be more a consequence of treatment failure than of the VAP itself. Surprisingly, those with ECMO-AI from other infection site such as BSI did not seem to have a poorer prognosis, which contrast with the available literature in patients without ECMO support [32, 33]. Exposure of the patient’s blood to the non-epithelialized surface of tubing induces early immune changes [5]. The activation of the innate immune system increased the odds of further secondary infection and subsequent immune response [3] and may finally impact patient’s outcome. Further studies are needed in order to better understand ECMO-AI impact in critically illness.

Surprisingly, patients cannulated in the more recent years of the study were at higher risk of AI. This unexpected result can be explained not only by modifications in indication for VV-ECMO cannulation, management, but also case mix, work overload and remaining confounders. Finally, patients with bacterial cause of ARDS had a lower risk of VAP than those with a viral etiology. Indeed, viral pneumonia may induce damages to the ciliated cells, decreasing mucociliary clearance and increasing risk for bacterial colonization and proliferation in the airways.

Our study is the first to assess the benefit of MSD in VV-ECMO patients. However, some limitations should be acknowledged. First, since this was a retrospective observational design and not a randomized control trial, residual cofounders may persist despite multivariable adjustment and propensity-score matching. Second, local practices regarding ECMO and ARDS patients’ management may differ such as strategy for VAP diagnosis and treatment or annual ECMO case volume, both variables being associated with patient’s outcome [34]. Third, in La Pitié Salpétrière cannulation was mainly performed in nearby ICUs by a mobile ECMO retrieval team before admission. However, patients managed by this skilled team have a similar complications rate, including ECMO-AI, than those cannulated directly in the referral center [35]. A randomized control trial should be design to go through this issue. Fourth, the adherence to the local protocol of VAP prevention was not retrieved. Finally, our study might be underpowered to study patient important outcome such as mortality.

## Conclusion

In this cohort of patients from different hospitals MSD appeared to be safe in ECMO patients and may be associated with improved outcomes including a decreased incidence of ECMO-AI. There were no differences in mortality rate in between groups, but fewer days with antimicrobial treatment and finally less MDRO acquisition among patients receiving MSD. These favorable results deserve confirmation by randomized controlled trials.

## Supplementary Information

**Additional file 1:** **Table S1.** Data regarding antimicrobial agent in each group. **Table S2.** Risk factors for death in ICU (logistic regression). **Table S3.** Baseline characteristics and outcomes of matched pairs.

## Data Availability

The datasets generated during the current study are available from the corresponding author on reasonable request.
