# Supplementary material for: Multiple-site decontamination to prevent acquired infection in patients with veno-venous ECMO support
Source: Ann Intensive Care. 2023 Apr 7;13:27. doi: 10.1186/s13613-023-01120-1 (PMC10079793; doi:10.1186/s13613-023-01120-1)
Supplement: Supplementary file 1 — Additional file 1: Table S1. Data regarding antimicrobial agent in each group. Table S2. Risk factors for death in ICU (logistic regression). Table S3. Baseline characteristics and outcomes of matched pairs. [file 13613_2023_1120_MOESM1_ESM.docx]

Table S1. Data regarding antimicrobial agent in each group.

| Variables | Missing data | SC | MSD | p-value |
| --- | --- | --- | --- | --- |
|  | SC/MSD | n = 172 | n = 69 |  |
| Proportion of length of ICU stay with antimicrobial agent, % | 0/10 | 86 [37-100] | 19 [2-53] | <0.001 |
| Number of day with antimicrobial treatment, days | 0/10 | 13 [5-30] | 7 [2-10] | <0.001 |
| Number of day alive without antimicrobial treatment at day 60 | 0/10 | 27 [0-53] | 42 [8-53] | 0.009 |
| *Treatment of AI* |  |  |  |  |
| Number of day with antimicrobial treatment for AI, days | 0/0 | 7 [0-14] | 0 [0-5] | <0.001 |
| Number of day alive without antimicrobial treatment for AI at day 60 | 0/0 | 41 [3-60] | 50 [16-60] | 0.012 |
| *Treatment of ARDS aetiology* |  |  |  |  |
| Number of day with antimicrobial treatment for ARDS aetiology, days | 0/10 | 6 [3-10] | 5 [2-8] | 0.213 |
| Number of day alive without antimicrobial treatment for ARDS aetiology at day 60 | 0/10 | 47 [4-54] | 46 [7-55] | 0.289 |
| Antimicrobial agent as treatment of ARDS aetiology |  |  |  |  |
| Amoxicillin and Clavulanic acid | 0/26 | 27 (16.9) | 7 (13.2) | 0.678 |
| 3rd-Generation Cephalosporin | 0/26 | 82 (51.2) | 29 (54.7) | 0.780 |
| Anti-Pseudomonas Cephalosporin | 0/26 | 17 (10.6) | 2 (3.8) | 0.215 |
| Piperacillin / Tazobactam | 0/26 | 51 (31.9) | 9 (17.0) | 0.056 |
| Carbapanem | 0/26 | 25 (15.6) | 1 (1.9) | 0.016 |
| Macrolides | 0/26 | 78 (48.8) | 19 (35.8) | 0.140 |
| Aminoglycoside | 0/26 | 14 (8.8) | 8 (15.1) | 0.291 |
| Quinolones | 0/26 | 23 (14.4) | 8 (15.1) | 1.000 |
| Others | 0/26 | 44 (27.5) | 9 (17.0) | 0.176 |
| Combination therapy | 0/26 | 150 (93.8) | 40 (75.5) | 0.001 |

Table S2. Risk factors for death in ICU (logistic regression).

| Variables | OR | 95%CI | p | OR | 95%CI | p-value |
| --- | --- | --- | --- | --- | --- | --- |
|  |  |  |  |  |  |  |
| Multiple site decontamination | 1.08 | 0.61-1.90 | 0.79 |  |  |  |
| Age, per supplementary year | 1.01 | 0.99-1.03 | 0.11 | 1.01 | 0.98-1.04 | 0.68 |
| Male | 1.05 | 0.61-1.78 | 0.87 |  |  |  |
| *Comorbidities* |  |  |  |  |  |  |
| Immunocompromised | 2.11 | 1.17-3.81 | 0.013 | 1.60 | 0.49-5.31 | 0.44 |
| Diabetes | 1.44 | 0.74-2.84 | 0.28 |  |  |  |
| Artery disease | 1.30 | 0.18-9.37 | 0.80 |  |  |  |
| Hypertension | 0.81 | 0.46-1.45 | 0.49 |  |  |  |
| Year of admission, per supplementary year | 0.97 | 0.87-1.09 | 0.64 |  |  |  |
| *ARDS etiology* |  |  |  |  |  |  |
| Viral | Ref | Ref | Ref |  |  |  |
| Bacterial | 1.44 | 0.70-2.98 | 0.33 |  |  |  |
| Others | 1.31 | 0.66-2.64 | 0.44 |  |  |  |
| MDRO colonization at admission | 0.61 | 0.26-1.42 | 0.25 |  |  |  |
| Non respiratory SOFA score, per supplementary point | 1.13 | 1.04-1.19 | 0.001 | 1.13 | 0.99-1.29 | 0.065 |
| PaO2/FiO2, per 1 point increment | 0.99 | 0.99-1.00 | 0.48 |  |  |  |
| PaCO2, per 1 point increment | 1.01 | 0.99-1.03 | 0.22 |  |  |  |
| Lactate, per 1 point increment | 1.11 | 0.99-1.25 | 0.087 | 1.02 | 0.88-1.18 | 0.87 |
| ECMO-associated infection | 1.71 | 1.00-2.95 | 0.049 | 1.09 | 0.35-3.39 | 0.87 |
| *Ventilator-associated pneumonia** | *1.84* | *1.05-3.23* | *0.033* | *1.61* | *0.49-5.24* | *0.42* |
| *Bloodstream infection** | *0.93* | *0.46-1.88* | *0.84* | *0.23* | 0.04-1.31 | 0.098 |

Note. *Each variables (ECMO associated infection, Ventilator-associated pneumonia and Bloodstream infection) were tested one by one in the multivariable analysis. ARDS : Acute respiratory distress syndrome. SOFA : sequential organ failure assessment. MDRO : Multidrug resistant micro-organisms. ICU : intensive-care unit. ECMO: extra corporeal membrane oxygenation.

Table S3. Baseline characteristics and outcomes of matched pairs.

| Variables | SC | MSD | Standardized mean diff | p-value |
| --- | --- | --- | --- | --- |
|  | n = 61 | n = 61 |  |  |
| Age, year | 50 [36-61] | 50 [34-64] | 0.00 | 0.414 |
| Male – no. (%) | 44 (72.1) | 42 (68.9) | 0.07 | 0.843 |
| *Comorbidities* |  |  |  |  |
| Immunocompromised– no. (%) | 16 (26.2) | 13 (21.3) | 0.04 | 0.671 |
| Diabetes– no. (%) | 9 (14.8) | 7 (11.5) | 0.1 | 0.789 |
| Artery disease– no. (%) | 2 (3.3) | 1 (1.6) | 0.03 | 1.000 |
| Hypertension– no. (%) | 14 (23.0) | 11 (18.0) | 0.13 | 0.654 |
| *Period of admission* |  |  | 0.01 | 1.00 |
| <2018 – no. (%) | 21 (67.2) | 42 (68.8) |  |  |
| 2018-2021 – no. (%) | 20 (32.8) | 19 (31.1) |  |  |
| *ARDS etiology* |  |  | 0.16 | 0.842 |
| Others – no. (%) | 22 (36.1) | 24 (39.3) |  |  |
| Bacterial – no. (%) | 21 (34.4) | 18 (29.5) |  |  |
| Viral – no. (%) | 18 (29.5) | 19 (31.1) |  |  |
| MDRO colonization at admission – no. (%) | 10 (16.4) | 5 (8.2) | 0.16 | 0.270 |
| SOFA score | 10 [8-14] | 9 [7-11] | 0.11 | 0.217 |
| PaO2/FiO2 at cannulation, mmHg | 69 [60-83] | 69 [54-87] | 0.16 | 0.848 |
| PaCO2 at cannulation, mmHg | 55 [48-76] | 54 [42-65] | 0.12 | 0.087 |
| Lactate at cannulation, mmol/L | 2.20 [1.30-3.50] | 2.40 [1.50-3.10] | 0.1 | 0.969 |
| *Outcomes* |  |  |  |  |
| ECMO-associated infection– no. (%) | 28 (45.9) | 13 (21.3) | - | 0.007 |
| Ventilator-associated pneumonia– no. (%) | 26 (42.6) | 11 (18.0) | - | 0.006 |
| Bloodstream infection– no. (%) | 11 (18.0) | 6 (9.8) | - | 0.296 |
| Number of day with antimicrobial treatment, days | 14 [6-32] | 7 [2-10] | - | <0.001 |
| Number of day alive without antimicrobial treatment at day 60 | 31 [0-53] | 42 [7-53] | - | 0.313 |
| Number of day alive and without treatment for AI at day 60 | 46 [9-60] | 50 [14-60] | - | 0.330 |
| Length of ECMO support, days | 13 [5-20] | 11 [5-22] | - | 0.792 |
| MDRO colonization acquisition – no. (%) | 11 (18.0) | 2 (3.3) | - | 0.019 |
| Death during ECMO support– no. (%) | 21 (34.4) | 23 (37.7) | - | 0.850 |
| Death in ICU – no. (%) | 23 (37.7) | 28 (45.9) | - | 0.36 |

Note. SC : standard care. MSD : Multiple site decontamination. ARDS : Acute respiratory distress syndrome. SOFA : sequential organ failure assessment. MDRO : Multidrug resistant micro-organisms. ICU : intensive-care unit. ECMO: extra corporeal membrane oxygenation.
